# Characteristics and clinical outcomes of patients with nonsmoking small cell lung cancer in Korea

**DOI:** 10.1186/s12890-022-01989-x

**Published:** 2022-05-18

**Authors:** Hye Seon Kang, Jung Uk Lim, Chang Dong Yeo, Chan Kwon Park, Sang Haak Lee, Seung Joon Kim, Korean Association for Lung Cancer, Korea Central Cancer Registry, Ho Cheol Kim, Chang Min Choi, Chi Young Jung, Deog Gon Cho, Jae Hyun Jeon, Jeong Eun Lee, Jin Seok Ahn, Yeongdae Kim, Yoo-Duk Choi, Yang-Gun Suh, Jung-Eun Kim, Young-Joo Won, Young-Chul Kim

**Affiliations:** 1 Division of Pulmonary and Critical Care Medicine, Department of Internal Medicine, Bucheon St. Mary’s Hospital, College of Medicine, The Catholic University of Korea, Seoul, Republic of Korea; 2 Division of Pulmonary and Critical Care Medicine, Department of Internal Medicine, Yeouido St. Mary’s Hospital, College of Medicine, The Catholic University of Korea, Seoul, Republic of Korea; 3 Division of Pulmonary and Critical Care Medicine, Department of Internal Medicine, Eunpyeong St. Mary’s Hospital, College of Medicine, The Catholic University of Korea, Seoul, Republic of Korea; 4 Division of Pulmonary and Critical Care Medicine, Department of Internal Medicine, Seoul St. Mary’s Hospital, College of Medicine, The Catholic University of Korea, 222, Banpo-daero, Seocho-gu, Seoul, 06591 Republic of Korea; 5 Department of Pulmonary and Critical Care Medicine, Asan Medical Center, College of Medicine, University of Ulsan, Seoul, Korea; 6 Department of Internal Medicine, Daegu Catholic University School of Medicine, Daegu, Korea; 7 Department of Thoracic and Cardiovascular Surgery, St. Vincent’s Hospital, College of Medicine, The Catholic University of Korea, Suwon, Korea; 8 Department of Thoracic and Cardiovascular Surgery, Seoul National University Bundang Hospital, Seoul National University College of Medicine, Seoul, Korea; 9 Division of Pulmonology Department of Internal Medicine, Chungnam National University, Daejeon, Korea; 10 Department of Medicine, Samsung Medical Center, Sungkyunkwan University, Seoul, Korea; 11 Department of Cardiothoracic Surgery, Pusan National University Hospital, Pusan, Korea; 12 Department of Pathology, Chonnam National University, Hwasun Hospital, Hwasun, Korea; 13 Proton Therapy Center, Research Institute and Hospital, National Cancer Center, Goyang, Korea; 14 Cancer Registration and Statistics Branch, National Cancer Center, Goyang, Korea; 15 Department of Internal Medicine, Chonnam National University, Hwasun Hospital, Hwasun, Korea

**Keywords:** Small cell lung carcinoma, Never-smokers, Korea

## Abstract

**Background:**

The aim of this study was to investigate the characteristics and clinical outcomes of patients with nonsmoking small cell lung cancer (SCLC) using a nationwide registry in Korea.

**Methods:**

The Korean Association for Lung Cancer developed a registry in cooperation with the Korean Central Cancer Registry (KCCR) and surveyed approximately 10% of recorded lung cancer cases.

**Results:**

From 2014 to 2016, the KCCR registered 1,043 patients newly diagnosed with SCLC among a total of 8,110 lung cancer patients. In subgroup analysis, Kaplan meier survival analysis showed that the overall survival (OS) was significantly shorter in the nonsmoking subgroup than the ever-smoking subgroup of SCLC patients with extensive disease (6.99 vs. 9.68 months; *P* = 0.016). Among SCLC patients with limited disease, OS was also shorter in the nonsmoking subgroup, without statistical significance (19.4 vs. 23.5 months; *P* = 0.247). In a multivariate analysis using a Cox regression model, never smoking was not associated with shorter OS, but older age, extensive stage, poor performance status (Eastern Cooperative Oncology Group grade ≥ 2), male sex, no prophylactic cranial irradiation, and no active treatment (chemotherapy and/or radiotherapy) were associated with poor prognosis.

**Conclusion:**

This evaluation of an unbiased nationwide survey dataset revealed that a significant proportion of Korean SCLC patients were never-smokers. No history of smoking appeared to be a significant prognostic factor according to the univariate analysis but was confirmed to be statistically insignificant through a multivariate analysis of the total population. Reasons for a poor prognosis may include the possibility that a high rate of the elderly population is composed of nonsmokers who did not receive active treatment.

## Background

Small cell lung cancer (SCLC) accounts for 10% to 15% of all lung cancers, although the incidence of SCLC has been declining with the decreasing prevalence of smoking [1]. SCLC is an aggressive malignancy with a short doubling time, high fraction ratio, and early development of distant metastasis [2]. SCLC is commonly viewed as a smoker’s disease and is very rare in those who have never been smokers [3]. In fact, nonsmoking-related SCLC may be a disease entity that is distinct from smoking-related SCLC. Recent research has found that significant differences exist regarding age distribution, sex, race, and mutational profiles between smokers and never-smokers with SCLC [4, 5]. Previous studies of prognosis have reported conflicting results, with some showing better survival among patients with SCLC who were never-smokers [3, 4, 6, 7], whereas others have reported no differences in survival [5].

To date, existing published data on SCLC in never-smokers largely originate from single-institution, retrospective studies [8–10]. Given the rarity of cases, a large, population-based study to investigate nonsmoking-related SCLC is warranted. We used the Korean Association for Lung Cancer (KALC) Registry (KALC-R), a nationwide unbiased registry developed by the KALC in cooperation with the Korean Central Cancer Registry (KCCR), and included about 10% of all lung cases in Korea [11].

In the present study, we retrospectively analyzed the clinical features and treatment strategies of nonsmoking SCLC patients and compared their clinical outcomes with those of smoking-related SCLC. Further, we identified independent predictors of survival among patients with SCLC. Stratifying SCLC subgroups based on smoking history may lead to treatment advances in managing this historically “recalcitrant” cancer.

## Materials and methods

### Study population and methods

Our study used data from the KALC-R, a database created using a retrospective sampling survey by the KCCR and the Lung Cancer Registration Committee [11]. Data from 13 regional cancer centers and 39 hospitals in Korea are included in this database, with the sample size of each hospital determined by the probability of selection according to the number of registrations. Patients were stratified by the date of diagnosis; sex; age; and their Surveillance, Epidemiology, and End Results program summary stage. Excluding multiple primary cancer patients, 2,621 patients in 2014, 2,660 patients in 2015, and 2,829 patients in 2016 were selected from the 52 centers through systematic sampling methods [12]. Of the total 8,110 patients registered between 2014 and 2016, those with no survival data and no smoking history were excluded, and 1,043 SCLC patients were selected. Finally, 154 never-smoker SCLC patients and 889 ever-smoker SCLC patients were compared and their data were analyzed to investigate differences between their clinical characteristics, treatment modalities, and clinical outcomes.

Based on a standardized protocol, the data of age, sex, body mass index (BMI), smoking history, symptoms, results of radiologic findings, Eastern Corporative Oncology Group (ECOG) performance status (PS) at the time of diagnosis, clinical stage, treatment modalities, and survival status were collected. The registered patients were followed until December 31, 2018 [12].

### Statistical analysis

Continuous variables are expressed as mean ± standard deviation or median (range) values and categorical variables are expressed as percentages. Continuous variables were compared using the Mann–Whitney U test, and categorical variables were compared using the chi-squared test. Risk factors for mortality were analyzed using Cox proportional hazards models. Survival was analyzed by the Kaplan–Meier method and compared by log-rank tests. All p-values were two-tailed, with statistical significance set at p < 0.05. All statistical analyses were performed using the Statistical Package for the Social Sciences version 20.0 (IBM Corp., Armonk, NY, USA).

## Results

### Prevalence of nonsmoking SCLC and patient characteristics by smoking history

**Fig. 1:**
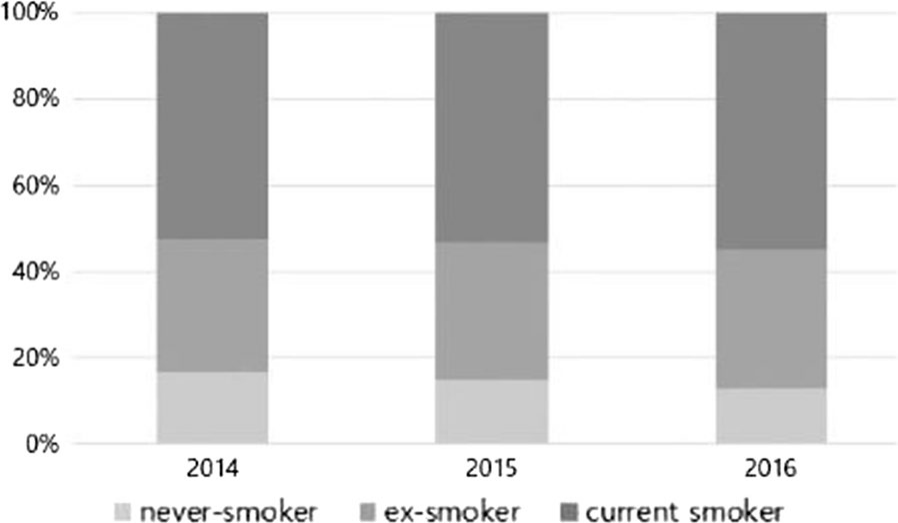
The distribution of smoking status in patients with small cell lung cancer by the year

The proportion of nonsmoking SCLC was steadily reduced from 2014 to 2016 (16.6% in 2014, 15.0% in 2015, and 12.7% in 2016) without statistically significance (*P* = 0.722) (Fig. 1). The median age of all study participants was 71 years. Never-smoking SCLC was more prevalent in women than in men (50.6% vs. 7.0%; *P* < 0.001). The proportion of extensive disease according to PS displayed a higher trend in the nonsmoking subgroup even though there was no statistically significant significance between those with and without a smoking history. Meanwhile, the proportions of receiving chemotherapy and treatment completeness were lower in the nonsmoking subgroup. The proportion of receiving best supportive care was not different between the two subgroups, but the receipt of local therapy only without chemotherapy was higher in the nonsmoking group (Table 1).

**Table 1:** Comparison of baseline characteristics between nonsmoking and smoking SCLC

	Never-smoker	Ever-smoker (current + ex)	*P* value
No of patients, %	154 (14.8%)	889 (85.2%)	
Age			
Median (range)	74 (35–91)	70 (32–96)	
≥ 65 years	126 (81.8%)	620 (69.7%)	0.002
Gender, female	78 (50.6%)	62 (7.0%)	< 0.001
BMI	23.28 ± 3.55	23.09 ± 3.35	0.534
Stage			0.115
Limited disease	47 (30.5%)	325 (36.6%)	
Extensive disease	102 (66.2%)	514 (57.8%)	
NA	5 (3.2%)	50 (5.6%)	
ECOG PS ≥ 2	24 (21.6%)	113 (16.8%)	0.212
Symptoms at the diagnosis	122 (79.2%)	673 (75.7%)	0.575
Treatment completeness	117 (76.0%)	744 (83.7%)	0.038
Treatment strategy			
Best supportive care	44 (28.6%)	220 (24.7%)	0.314
Chemotherapy	72 (46.8%)	516 (58.0%)	0.009
Chemotherapy and local treatment	15 (9.7%)	121 (13.6%)	0.188
Local therapy only	13 (8.4%)	32 (3.6%)	0.006
NA	10 (6.5%)	0 (0.0%)	

*SCLC* small cell lung cancer; *BMI* body mass index; *ECOG PS* Eastern Cooperative Oncology Group performance status; *NA* not available

The total number of cycles of initial chemotherapy was not different between the two groups, and there was no differences in regimen of initial chemotherapy between two groups (Table 2).

**Table 2:** Treatment strategies between nonsmoking and smoking SCLC patients

	Never-smoker	Ever-smoker (current + ex)	*P* value
Total number of cycles of 1st line chemotherapy			0.506
One	12 (13.8%)	80 (12.6%)	
Two	12 (13.8%)	64 (10.0%)	
≥ Three	63 (72.4%)	493 (77.4%)	
Regimen of initial chemotherapy			
Cisplatin + etoposide	45 (51.7%)	370 (58.1%)	0.261
Carboplatin + etoposide	32 (36.8%)	192 (30.1%)	0.209
Cisplatin + irinotecan	3 (3.4%)	28 (4.4%)	0.682
Irinotecan mono	3 (3.4%)	21 (3.3%)	0.529
Others	4 (4.8%)	26 (4.2%)	0.818

*SCLC* small cell lung cancer

At the diagnosis of SCLC, the proportion of patients with one or more symptoms was not different between the two groups, but those with hemoptysis (*P* = 0.063) or weight loss (*P* = 0.023) were less common in the never-smoker group. Radiologic findings such as mass diameter and structural invasion were similar, with the exception of recurrent laryngeal nerve invasion (Table 3).

**Table 3:** Symptoms and radiologic findings according to smoking status

	Never-smoker	Ever-smoker (current + ex)	*P* value
Symptoms at the diagnosis	122 (79.2%)	673 (75.7%)	0.575
Cough	62 (40.3%)	401 (45.1%)	0.264
Sputum	36 (23.4%)	244 (27.4%)	0.293
Dyspnea	54 (35.1%)	266 (29.9%)	0.201
Hoarseness	5 (3.2%)	35 (3.9%)	0.680
Hemoptysis	4 (2.6%)	57 (6.4%)	0.063
Weight loss	5 (3.2%)	76 (8.5%)	0.023
Radiologic findings			
The largest diameter	4.92 ± 2.54	4.98 ± 2.49	0.807
Obstructive pneumonia	29 (19.0%)	168 (18.9%)	0.427
Chest wall invasion	3 (1.9%)	30 (3.4%)	0.350
Diaphragm invasion	3 (1.9%)	11 (1.2%)	0.479
Pleural invasion	31 (20.1%)	127 (14.3%)	0.062
Pericardium invasion	6 (3.9%)	20 (2.2%)	0.226
Mediastinum invasion	27 (17.5%)	152 (17.1%)	0.895
Heart invasion	1 (0.6%)	8 (0.9%)	0.756
Trachea invasion	1 (0.6%)	16 (1.8%)	0.298
Esophagus invasion	1 (0.6%)	13 (1.5%)	0.418
Vertebra invasion	1 (0.6%)	7 (0.8%)	0.856
Great vessel invasion	25 (16.2%)	170 (19.1%)	0.396
Phrenic nerve invasion	0 (0.0%)	1 (0.1%)	0.677
Recurrent laryngeal nerve invasion	2 (1.3%)	2 (0.2%)	0.047
Cervical sympathetic	0 (0.0%)	1 (0.1%)	0.677
Main bronchus invasion	14 (9.2%)	129 (14.5%)	0.006

### Prognostic factors and survival analysis

**Fig. 2:**
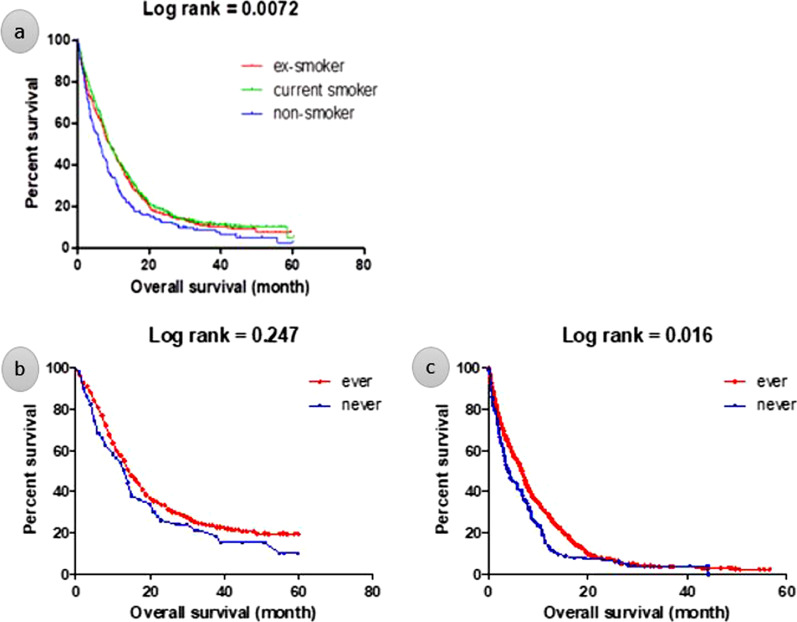
Kaplan–Meier Curve for overall survival according to smoking status in **a** total population, **b** limited SCLC and **c** extensive SCLC. *SCLC* small cell lung carcinoma

Overall survival (OS) was significantly shorter in the nonsmoking SCLC subgroup (11.03 vs. 15.15 vs. 14.30 months; *P* < 0.01) than in the current and ex-smoker subgroups. During subgroup analysis, OS was found to be shorter in the nonsmoking subgroup than in the ever-smoking subgroup of extensive-disease (ED)-SCLC patients (6.99 vs. 9.68 months; *P* = 0.016), but the OS was not different between the nonsmoking and ever-smoking subgroups among limited-disease (LD)-SCLC patients (19.4 vs. 23.5 months; *P* = 0.247) (Fig. 2).

In a multivariate analysis using a Cox regression model, never smoking was not associated with OS, but older age, extensive stage, poor PS (ECOG grade ≥ 2), male sex, no prophylactic cranial irradiation (PCI), and no active treatment (chemotherapy and/or radiotherapy) were associated with poor prognosis (Table 4).

**Table 4:** Univariate and multivariate analysis of clinical parameters on overall survival in patients with SCLC in total population

Variables		Univariate analysis		Multivariate analysis	
	No. of cases	HR (95% CI)	*P* value	HR (95% CI)	*P* value
Age	1,043	1.041 (1.034–1.049	< 0.001	1.020 (1.002–1.038)	0.033
Stage, ED	616	2.448 (2.121–2.825)	< 0.001	1.952 (1.398–2.727)	< 0.001
Never smoker	154	1.338 (1.119–1.599)	0.001	1.277 (0.676–2.409)	0.451
ECOG PS					
2–4 vs. 0–1	144 vs. 655	2.406 (1.987–2.914)	< 0.001	3.219 (1.860–5.572)	< 0.001
Gender, male	916	1.150 (0.950–1.392)	0.151	2.247 (1.172–4.305)	0.015
Symptom at diagnosis	82	1.902 (1.466–2.467)	< 0.001	1.659 (0.969–2.839)	0.065
PCI	70	0.336 (0.261–0.431)	< 0.001	0.606 (0.403–0.910)	0.016
Active-treatment (chemo and/or TRT)	724	0.408 (0.356–0.468)	< 0.001	0.291 (0.131–0.644)	0.002

*SCLC* small cell lung cancer; *HR* hazard ratio; *CI* confidence interval; *ED* extensive disease; *ECOG PS* Eastern Cooperative Oncology Group Performance status; *PCI* prophylactic cranial irradiation; *TRT* thoracic radiotherapy

We further performed subgroup analysis according to disease status. In the subgroup analysis of LD-SCLC, never smoking was significantly associated with poor OS after adjusting confounding factors. Also, symptoms observed at diagnosis were independently associated with poor prognosis, while treatment of PCI or chemotherapy was independently associated with favorable prognosis. In ED-SCLC patients, older age, poor PS, and brain or liver metastasis were independently associated with poor prognosis, but smoking status was not associated with clinical outcomes (Table 5).

**Table 5:** Univariate and multivariate analysis of clinical parameters on overall survival in patients with LD and ED-SCLC

		LD				ED			
Factor	No. of cases	Univariate analysis		Multivariate analysis		Univariate analysis		Multivariate analysis	
		HR (95% CI)	*P* value	HR (95% CI)	*P* value	HR (95% CI)	*P* value	HR (95% CI)	*P* value
Age	1,043	1.049 (0.133–0.064)	< 0.001	1.007 (0.983–1.031)	0.595	1.040 (1.030–1.050)	< 0.001	1.036 (1.009–1.064)	0.008
Gender (male)	916	1.215 (0.873–1.691)	0.249	1.863 (0.844–4.111)	0.124	0.968 (0.763–1.228)	0.786	2.496 (0.650–9.585)	0.183
Never smoking	154	1.260 (0.899–1.766)	0.180	2.410 (1.012–5.697)	0.047	1.348 (1.086–1.675)	0.007	1.392 (0.428–4.530)	0.582
ECOG PS(≥ 2)	144	2.306 (1.612–3.300)	< 0.001	2.408 (0.984–5.894)	0.054	2.623 (2.073–3.318)	< 0.001	3.244 (1.543–6.820)	0.002
Symptom at diagnosis	82	1.914 (1.302–2.812)	0.001	2.082 (1.023–4.237)	0.043	1.357 (0.939–1.959)	0.104	1.215 (0.512–2.883)	0.659
PCI	70	0.463 (0.296–0.726)	0.001	0.527 (0.320–0.868)	0.012	0.798 (0.427–1.489)	0.478	1.131 (0.517–2.477)	0.758
Chemo + TRT	136	0.467 (0.359–0.608)	< 0.001	0.472 (0.295–0.755)	0.002	0.356 (0.196–0.648)	0.001	0.044 (0.009–0.220)	< 0.001
Chemo only	588					0.462 (0.389–0.548)	< 0.001	0.069 (0.018–0.273)	< 0.001
Pleural effusion	173					2.062 (1.356–3.136)	< 0.001	1.831 (1.005–3.336)	0.048
Pleural nodules	74					1.334 (1.029–1.731)	0.030	1.167 (0.460–2.959)	0.745
Bone meta	260					1.373 (1.166–1.616)	< 0.001	1.544 (0.993–2.402)	0.054
Brain	184					0.897 (0.753–1.069)	0.223	1.675 (1.067–2.630)	0.025
Liver meta	219					1.728 (1.457–2.049)	< 0.001	2.251 (1.368–3.705)	0.001
Adrenal meta	104					1.128 (0.908–1.401)	0.276	1.648 (0.858–3.168)	0.134

*LD* limited disease; *ED* extensive disease; *HR* hazard ratio; *CI* confidence interval; *ECOG* Eastern Cooperative Oncology Group Performance status; *PCI* prophylactic cranial irradiation; *TRT* thoracic radiotherapy

## Discussion

Our study indicated that never-smokers are prevalent in SCLC in Korea. During the study period, the prevalence of never-smoking SCLC was steadily reduced, without statistical significance. The proportions of female sex and elderly age were significantly higher in the never-smoking SCLC group. We also found that ever-smokers were more likely to receive chemotherapy and/or radiotherapy, while never-smokers were more likely to receive radiotherapy only. The proportions of ED-SCLC and poor PS exhibited a higher trend among never-smokers even though there was no statistical significance. Clinical symptoms such as hemoptysis or weight loss were more frequently demonstrated in the ever-smoker group than the never-smoker group. In Kaplan Meier survival analysis, never-smoking SCLC patients had significantly shorter OS periods relative to ever-smokers in both the total study population and ED-SCLC subgroup. However, never smoking was significantly associated with poor OS in LD-SCLC but not in ED-SCLC patients after adjusting confounding factors. Meanwhile, older age, ED, poor PS, male sex, and not receiving PCI or active treatment such as chemotherapy and/or radiotherapy were significantly associated with a shorter OS in the total population.

In our study, the prevalence of nonsmoking SCLC was 14.8%, which is higher than that reported in non-Asian countries and contradicts the traditional belief that SCLC is a smoker’s disease. The prevalence of never-smoker SCLC was reported to be only 2.5% to 3.4% in non-Asian countries [3, 13]. In contrast, some studies carried out in Asia revealed a greater incidence of cases. In Chinese populations, the proportion of never-smoking SCLC was 22.8%, which is higher than our results [6]. Our findings are in line with those of a previous independent study reporting a high prevalence (about 13.0%) of never-smokers among SCLC patients in Korea [7, 14]. The high proportion of never-smokers among Asian lung cancer patients could not be explained exactly but is suggested to be attributed to ethnic differences. Also, secondhand smoking status, occupational carcinogen exposure, and other important risk factors involved in carcinogenesis could not be analyzed in our study [15]. Finally, the proportion of elderly patients was higher in the never-smoker subgroup in our study. The average annual growth in the aging rate in Korea is 3.3%, which is the fastest rate among 37 OECD countries. Korea has had an aging society since 2000 and is expected to demonstrate an ultra-aged society in 2026 [16]. Lung cancer is an aging-related disease, and nonsmoking SCLC is thought to occur in the elderly by accumulating exposure to other environmental factors, regardless of smoking, in an aging society [17].

Previous studies of prognosis have reported conflicting results. Some studies demonstrated that never-smokers with SCLC had a better prognosis than that of smokers with SCLC [3, 4, 6, 7]. Researchers hypothesized that this phenomenon may be partly attributed to the fact that never-smokers have fewer comorbidities and can better tolerate the treatment [18]. Other studies have reported no survival differences between nonsmoking and smoking SCLC patients [5]. In our study, never smoking appeared to be a significantly poor prognostic factor according to the univariate analysis but was confirmed to be statistically insignificant through a multivariate analysis of the total population. However, nonsmoking SCLC had a significantly poor prognosis relative to smoking SCLC in LD patients in the multivariate analysis. Contrary to as predicted by nonsmoking, the reasons for a poor prognosis may include the possibility of a high number of elderly patients among nonsmokers who did not receive active treatment and because the proportion of treatment completeness was lower.

Elderly SCLC patients are difficult to treat by standard methods for many reasons; for example, they often have multiple comorbidities and poor PS. Among patients diagnosed with SCLC, about 43% are 70 years of age or older and 10% are 80 years of age or older [19]. In elderly SCLC patients with good PS, platinum-based chemotherapy plus thoracic radiotherapy and carboplatin-based chemotherapy are recommended for LD- and ED-SCLC, respectively. Even when patients are old and have poor PS, treatment with chemotherapy is suggested if the poor PS is due to SCLC [20]. In our study, the proportions of treatment completeness and receiving chemotherapy were lower and that of receiving local therapy only was higher in the nonsmoking group, which had a higher proportion of elderly patients. Our findings are in line with those of a previous study where patients aged 70 years or younger were treated with best supportive care in only 17% to 23% of cases, but the percentage increased with higher age up to 75% of those aged 85 years or older [21]. Elderly SCLC patients experienced more severe adverse events, completed treatment less often, and died during treatment more frequently than younger patients [22]. However, active treatment, including chemotherapy and/or radiotherapy, has improved the survival of SCLC patients younger than 80 years of age [21]. It is necessary to consider active treatment in elderly SCLC patients who are not very old, taking into account their general condition.

The prognosis of never-smoking SCLC is known to be better than that of ever-smoking SCLC, even though a recent study reported the prognosis is not different between the two groups [5–7]. Further, radon-related SCLC, one type of nonsmoking SCLC, demonstrates aggressive features [23]. The exact molecular mechanisms of nonsmoking SCLC are not known; for example, different molecular signatures might exist at MEK and mTOR pathways [24]. Another theory is that SCLC is phenotypically transformed from pulmonary adenocarcinoma with epidermal growth factor receptor mutation (EGFR) mutations as an acquired-resistance mechanism during EGFR-tyrosine kinase inhibitor treatments [25, 26]. Among 28 genetically evaluable nonsmoking SCLC patients in Korea, EGFR mutations were detected in four cases [7]. Further investigation of relevant genetic and environmental factors in the context of never-smoking SCLC is needed.

Previous studies have reported conflicting results regarding the association between smoking status and age [27, 28]. In our study, ever smokers were significantly younger and had a slightly more favorable LD-SCLC than never-smokers did. These findings could be explained by that more frequent screenings for lung cancer in former smokers resulted in the detection of early-stage lung cancer [18]. Ever-smokers with less advanced disease and younger age showed more favorable OS outcomes in our study.

The substantial prevalence of SCLC among never-smokers is not explained conclusively, but there is evidence to suggest that documentation of smoking status is varied and can differ between various studies. Smoking history is subject to recall biases and self-reported reliability, and there may be a small misclassification of smokers as nonsmokers [27]. Other patients with risk factors for SCLC, such as exposure to environmental tobacco smoking, some work-related fumes, and indoor radon, might be included in the never-smoking SCLC group even though we could not analyze these risk factors in our study because the data collection did not consider these variables [23]. Of the types of nonsmoking SCLC, indoor radon-related SCLC is an aggressive type, and age at diagnosis is higher for histologic types other than this type in never-smokers [29].

One of the problems with SCLC is a late diagnosis, and presenting symptoms at the time of diagnosis can indicate a poor prognosis [23]. Contrary to other histologic tumor types, the central location of SCLC could cause symptoms earlier on and lead to early detection in the localized stages of the disease. In our study, the proportions of patients having one or more symptoms at the time of diagnosis were not similar but hemoptysis and weight loss were more frequently found in the ever-smoker group. It is estimated that nonsmokers with less symptoms who do not receive regular screening could receive a lung cancer diagnosis at a late age and carry a poorer prognosis.

This study had several limitations. First, this was a retrospective study lacking information such as secondhand smoking status and comorbidities. Also, due to the structure of the data, progression-free survival could not be confirmed, so there is a limitation in not confirming the relationship between smoking status and disease control after treatment. However, our study also had strengths given its use of an unbiased sampling method to sample a representative population of patients with lung cancer using a nationwide survey. Also, the number of patients in this study was relatively high. Our study will help broaden the understanding of the current epidemiologic status of SCLC and clinical characteristics in Korea.

## Conclusions

In conclusion, this evaluation of an unbiased nationwide survey dataset revealed that a significant proportion of Korean SCLC patients were never-smokers. Never smoking appeared to be a significant prognostic factor according to the univariate analysis but was confirmed to be statistically insignificant through a multivariate analysis of the total population. These patients were older and showed a tendency not to receive active treatments. Active treatment of SCLC in aged patients not older than 80 years can improve survival, so a better understanding of the impact of treatment and toxic effects would enable physicians to discuss the risks and benefits of treatment with never-smoking patients.

## Data Availability

The datasets used and/or analyzed during the current study have been kept confidential and are not available publicly because the KALC and the Ministry of Health and Welfare, KCCR do not allow researchers to provide data personally or share publicly, but are available from the corresponding author upon reasonable request.

## Abbreviations

SCLC: Small cell lung cancer
KCCR: The Korean Central Cancer Registry
OS: Overall survival
KALC: The Korean Association for Lung Cancer
KALC-R: The Korean Association for Lung Cancer Registry
KCCR: The Korean Central Cancer Registry
BMI: Body mass index
ECOG: Eastern Corporative Oncology Group
PS: Performance status
ED: Extensive-disease
LD: Limited-disease
PCI: Prophylactic cranial irradiation
HR: Hazard ratio
TRT: Thoracic radiotherapy
CI: Confidence interval
EGFR: Epidermal growth factor receptor mutation
